# Lenalidomide-Associated Liver Injury With Features of Vanishing Bile Duct Syndrome in Multiple Myeloma

**DOI:** 10.7759/cureus.91775

**Published:** 2025-09-07

**Authors:** Sharvani Alajpur, Aamer Farooq

**Affiliations:** 1 Department of Internal Medicine, Saint Peter's University Hospital, New Brunswick, USA; 2 Department of Hematology and Medical Oncology, Saint Peter's University Hospital, New Brunswick, USA

**Keywords:** drug-induced liver injury, elevated liver transaminases, lenalidomide, multiple myeloma, vanishing bile duct syndrome

## Abstract

We report a rare case of lenalidomide-associated hepatotoxicity with histological features suggestive of vanishing bile duct syndrome (VBDS) picture in a woman with multiple myeloma (MM). A 67-year-old woman presented with a three-day history of productive cough, sore throat, fatigue, and pruritus. She has high-risk MM with vertebral metastasis and cord compression, previously treated with palliative radiation. She had received two cycles of treatment with lenalidomide, bortezomib, dexamethasone, and daratumumab, initiated five weeks prior to presentation. She had no history of alcohol use or viral hepatitis. The exam showed scleral icterus and fine left basilar crackles. Labs revealed pancytopenia, hyperbilirubinemia, and elevated liver enzymes. Testing for viral hepatitis and autoimmune markers was negative. Low levels of gamma globulins on serum protein electrophoresis ruled out infiltrative myeloma. Ultrasound and magnetic resonance imaging of the liver were unremarkable. A liver biopsy showed portal fibrosis with obliteration and herniation of portal vein branches, focal mild periductal fibrosis, and mild inflammation of the portal tracts, with some small portal tracts demonstrating missing or atrophic bile ducts. The patient was treated with dexamethasone 4 mg twice daily and ursodeoxycholic acid 600 mg twice daily with serial monitoring of liver function tests (LFTs). Left lower lobe pneumonia was confirmed on chest X-ray and was treated with antibiotics. One week prior, her LFTs were normal. Although aminotransferases downtrended with treatment, bilirubin rose, reaching 13.8 mg/dL. She was discharged on dexamethasone 4 mg daily and ursodeoxycholic acid 600 mg twice daily. Complete bilirubin normalization occurred three months after the discontinuation of lenalidomide when she was restarted on treatment for MM with bortezomib. She is currently being followed up at Saint Peter's Cancer Center with persistent elevation of alkaline phosphatase (ALP). The patient experienced grade 3 hepatobiliary toxicity as per the Common Terminology Criteria for Adverse Events (CTCAE) v5.0. This case highlights VBDS as a manifestation of lenalidomide-induced liver injury with persistent elevation of ALP and histologic findings of bile duct loss in small portal tracts, possibly representing a partial or early form of the condition. Early recognition of this potential hepatotoxicity pattern may guide timely drug discontinuation and appropriate management.

## Introduction

Multiple myeloma (MM) is a clonal plasma cell proliferative disorder characterized by the abnormal increase of monoclonal immunoglobulins leading to evidence of specific end-organ damage [1]. Lenalidomide is an immunomodulatory and anti-neoplastic drug effective in MM [2]. Non-hematologic toxicities like drug-induced liver injury (DILI) are rare compared to hematologic toxicities like myelosuppression. Hepatotoxicity associated with lenalidomide is uncommon but clinically significant, with only a few cases documented in the literature. While most cases are mild, severe hepatic injury can occur. The reported incidence of serious hepatic adverse effects is less than 1% in clinical trials, while mild, self-limited elevations in serum enzymes are observed in approximately 8-15% of patients [3,4]. DILI can present with variable histopathologic patterns, with inflammatory cell infiltration being the most reported finding in the literature. We report a rare case of lenalidomide-associated hepatotoxicity with vanishing bile duct syndrome (VBDS)-like picture with ductopenia in a woman with MM.

VBDS is a rare condition characterized by the progressive loss, destruction, and disappearance of the intrahepatic bile ducts, leading to cholestasis and ductopenia. Drug-induced VBDS is particularly significant, accounting for 7% of all VBDS cases [5].

## Case presentation

A 67-year-old African American woman presented with a three-day history of productive cough, sore throat, and fatigue. She also had new-onset pruritus. Medical history is significant for a high-risk MM (IgG 2771 mg/dL, 4:14 translocation) with vertebral metastasis and T2-T4 cord compression, treated with palliative radiation. She had received two cycles of treatment with lenalidomide, bortezomib, dexamethasone, and daratumumab, initiated five weeks prior to presentation. She had no history of alcohol use or viral hepatitis. Physical exam showed normal vitals, scleral icterus, and fine left basilar crackles. 

Labs revealed pancytopenia and elevated bilirubin, alkaline phosphatase (ALP), alanine aminotransferase (ALT), and aspartate aminotransferase (AST) levels, as shown in Table 1. Low levels of gamma globulins ruled out infiltrative myeloma. Testing for viral hepatitis and autoimmune markers (anti-nuclear antibody, anti-mitochondrial antibody, anti-smooth muscle antibody) was negative. Cytomegalovirus (CMV) PCR was positive, but CMV IgM was negative, and there was no evidence of CMV hepatitis on histopathology. Chest X-ray showed left lower lobe pneumonia. Ultrasound and magnetic resonance imaging (MRI) of the liver were unremarkable.

**Table 1:** Laboratory findings WBC: white blood cell; ALP: alkaline phosphatase; AST: aspartate aminotransferase; ALT: alanine aminotransferase; INR: international normalized ratio; LDH: lactate dehydrogenase; CRP: C-reactive protein; SPEP: serum protein electrophoresis

Lab test	Units	Result	Reference range
Hemoglobin	g/dL	10.9	12.0-16.0
WBC	×10³/µL	2.1	4.0-11.0
Neutrophils	×10³/µL	1.51	2.0-7.5
Lymphocytes	×10³/µL	0.44	1.0-3.5
Platelet count	×10³/µL	119	150-400
Total bilirubin	mg/dL	7.4	0.1-1.2
ALP	U/L	363	53-141
AST	U/L	507	14-36
ALT	U/L	531	0-35
INR	-	1.1	NA
Total protein	g/dL	5.1	6.0-8.0
Albumin	g/dL	3	3.2-4.6
LDH	U/L	682	140-271
CRP	mg/L	42.1	<10
Serum IgA	mg/dL	<40	70-400
Serum IgG	mg/dL	428	700-1600
Serum IgM	mg/dL	32	40-230
Gamma globulins (SPEP)	g/dL	0.36	0.6-1.5

**Figure 1:**
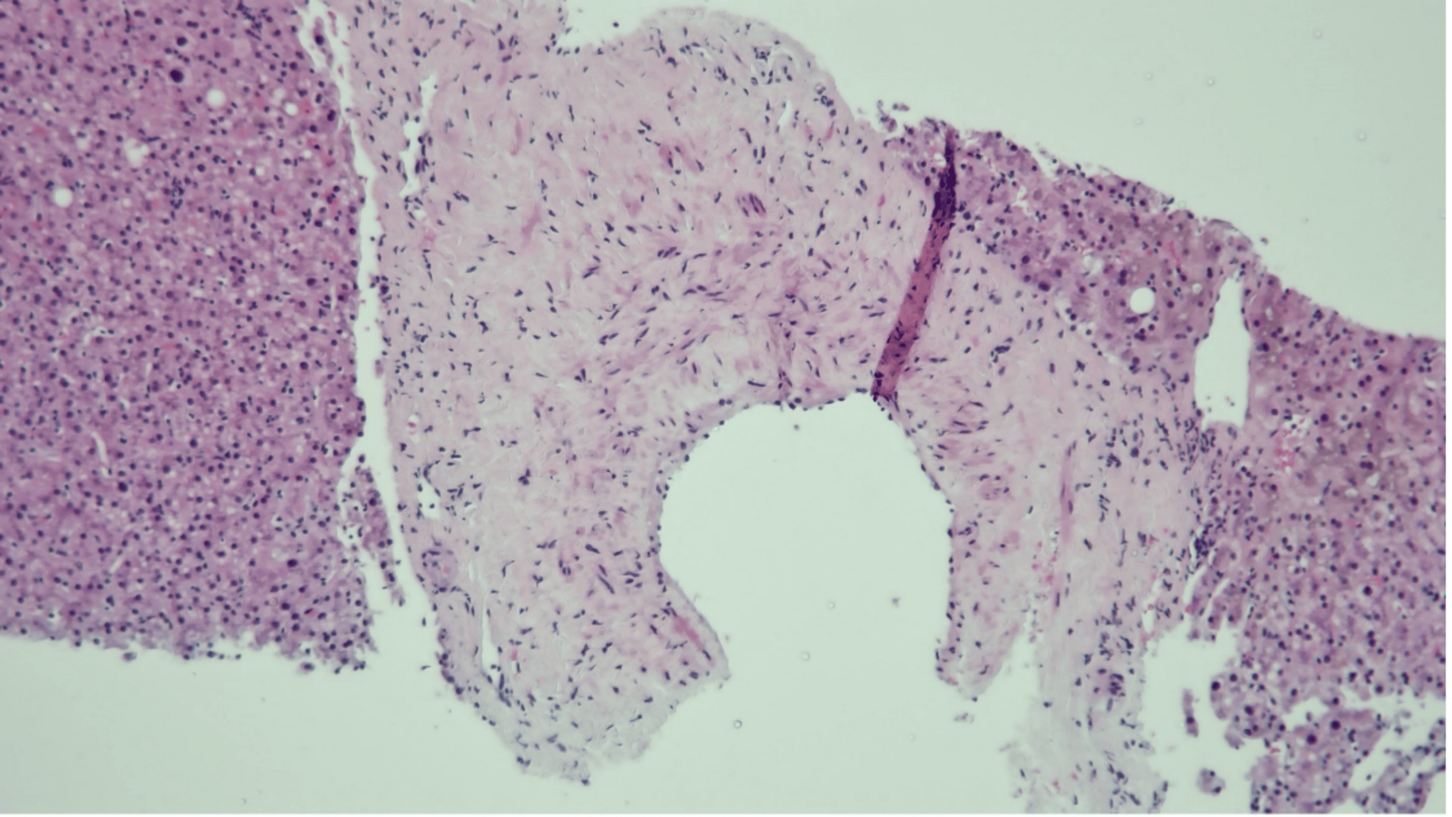
Liver biopsy showing the portal tract filled with fibrous stroma and inflammatory cells (hematoxylin and eosin, original magnification ×200)

**Figure 2:**
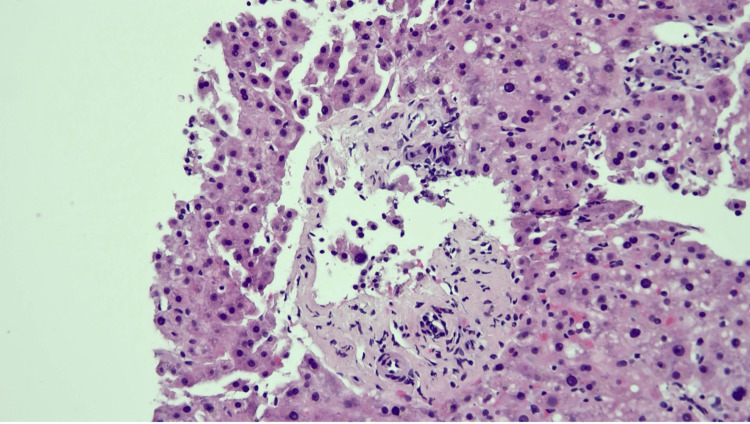
Liver biopsy showing the obliteration and herniation of portal vein branches, portal inflammation, and loss of small bile ducts (hematoxylin and eosin, original magnification ×200)

**Figure 3:**
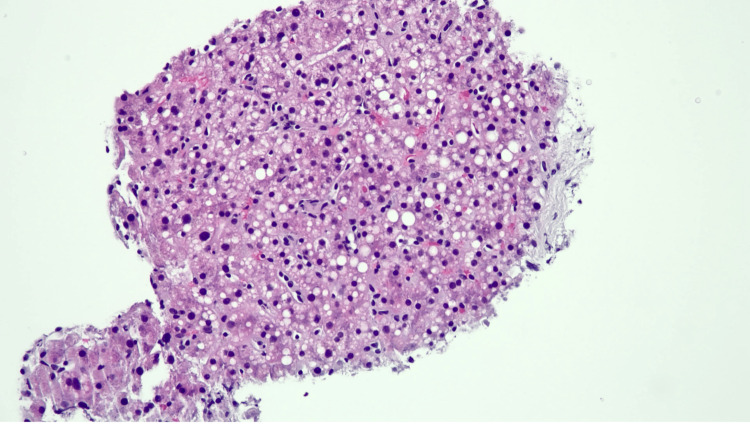
Liver biopsy showing mild lobular inflammation and steatosis (hematoxylin and eosin, original magnification ×200)

An ultrasound-guided liver biopsy performed on day 7 of hospitalization revealed portal fibrosis with obliteration and herniation of portal vein branches, focal mild periductal fibrosis, and mild inflammation of the portal tracts, with some small portal tracts demonstrating missing or atrophic bile ducts (Figures 1-2). Mild steatosis, hepatocellular cholestasis, and mild lobular necroinflammation were also present (Figure 3).

After ruling out other causes of liver injury, a diagnosis of DILI was established, supported by the histopathological findings. The Roussel Uclaf Causality Assessment Method (RUCAM) score, a tool used to assess the likelihood that liver injury is caused by a drug, was 9, indicating a high probability of DILI.

**Figure 4:**
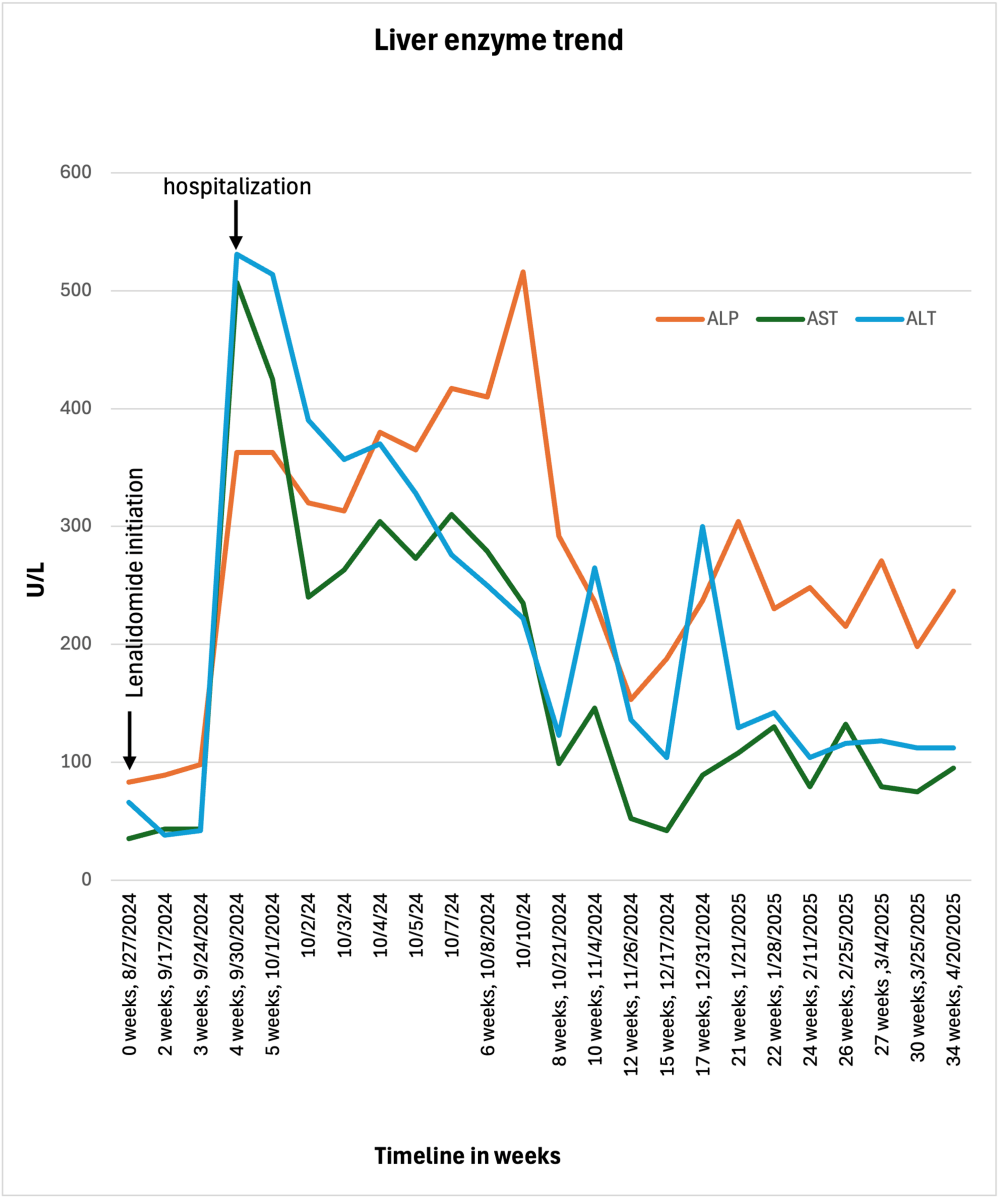
ALP and aminotransferase levels downtrended over a six-month course after the discontinuation of lenalidomide. Persistent ALP elevation was noted even after six months of drug discontinuation ALP: alkaline phosphatase

**Figure 5:**
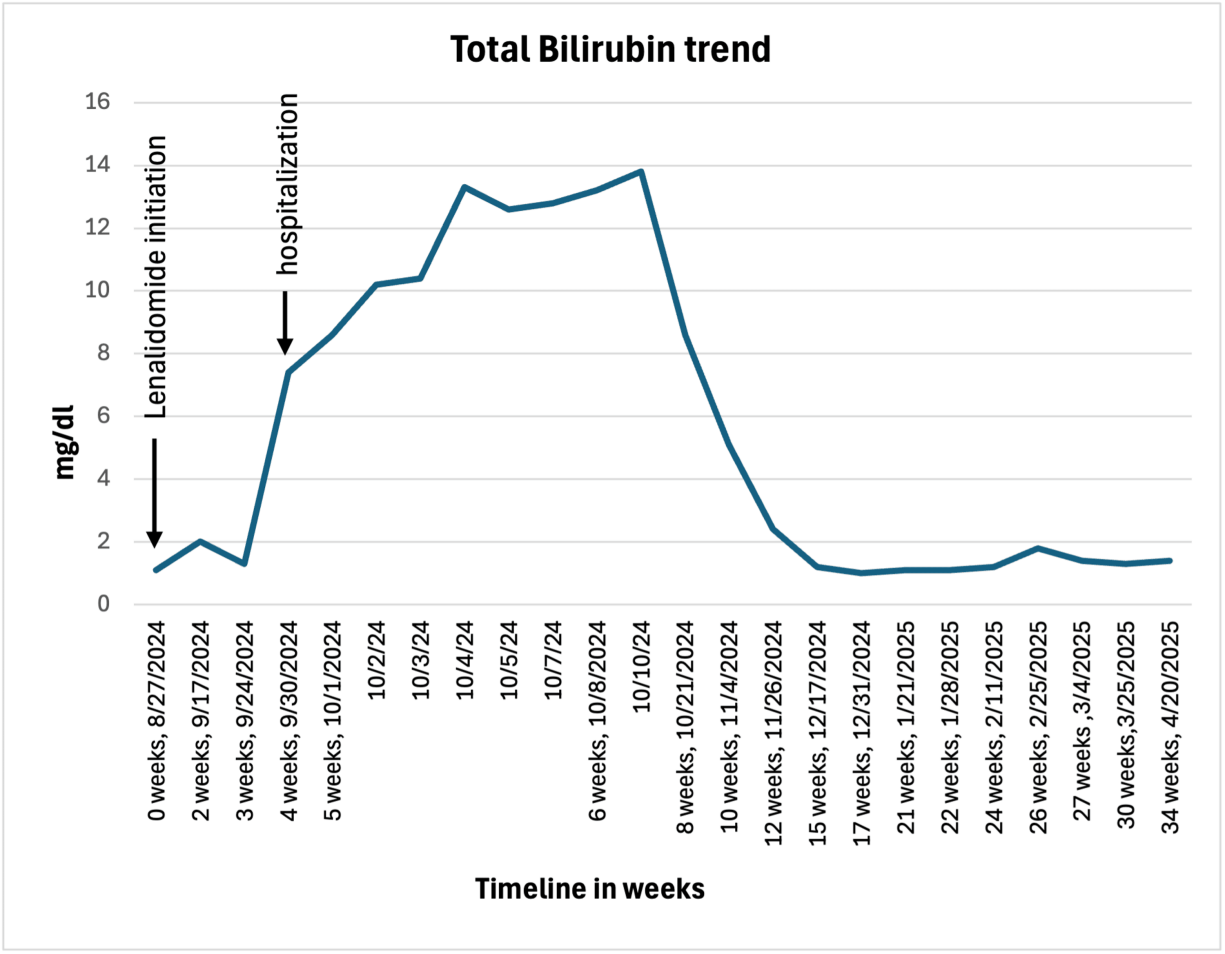
Bilirubin levels increased over the first 10 days of hospitalization and gradually decreased with treatment and normalized after three months

With DILI being the most likely diagnosis, the patient was treated with dexamethasone 4 mg twice daily and ursodeoxycholic acid 600 mg twice daily with serial monitoring of liver function tests (LFTs). Her LFTs were normal one week before the presentation. Although aminotransferases trended down with treatment, bilirubin rose, reaching 13.8 mg/dL (normal range: 0.1-1.2 mg/dL) over the 10-day hospital course (Figures 4-5). Additionally, the patient was treated with cefepime 2 g every eight hours for five days and azithromycin 500 mg daily for three days for community-acquired pneumonia in the setting of an immunocompromised state. She was discharged on dexamethasone 4 mg daily and ursodeoxycholic acid 600 mg twice daily, with plans to restart MM treatment after the normalization of LFTs. Complete bilirubin normalization occurred three months after the discontinuation of lenalidomide when she was restarted on treatment for MM with bortezomib. She is currently being followed up at Saint Peter's Cancer Center with persistent elevation of ALP.

## Discussion

Lenalidomide is classified as hepatotoxic category C (probable rare cause of clinically apparent liver injury) based on the number of cases reported, though DILI cases are often underreported. There have been prior case reports of lenalidomide-associated liver injury with hepatocellular inflammation on liver biopsy. We noted one case report of VBDS in a patient with MM treated with lenalidomide [6]. Compared to our case, the patient experienced a more severe liver injury that necessitated plasmapheresis and resulted in death four months after the initial presentation. The authors suppose the combination of bortezomib and lenalidomide may have a role in the development of VBDS.

The mechanism of drug-induced ductopenia may involve immunologically mediated bile duct damage, direct drug or reactive metabolite toxicity, or alterations in bile composition, impairing the bile ducts' protective functions against bile salt cytotoxicity. The extent of ductopenia varies, determined by the proportion of portal tracts lacking interlobular ducts. Severe ductopenia is defined as a reduction of at least 50% of portal tracts or a ratio below 0.5 (normal typically ranges from 0.9 to 1.8), though less severe cases can also occur [7].

The clinical outcomes of drug-induced VBDS vary widely, ranging from gradual and complete recovery after the discontinuation of the drug in some cases to progression to liver failure and death in others. Factors such as the degree of bile duct loss and histological findings, including advanced fibrosis, may predict poor prognosis [8]. While many cases of DILI are reversible, some may develop progressive liver injury leading to cirrhosis even after the offending drug is stopped.

In our case, the patient presented with pruritus, elevated liver enzymes, and increased bilirubin levels. The calculated R-factor was 4.4, consistent with a mixed hepatocellular and cholestatic pattern of injury. She developed grade 3 hepatobiliary toxicity, as defined by the National Cancer Institute Common Terminology Criteria for Adverse Events (CTCAE) v5.0, necessitating the discontinuation of lenalidomide [9].

A comprehensive evaluation by a multidisciplinary team was undertaken to rule out alternative causes of liver injury, including infections, autoimmune diseases, and malignancy-related hepatic involvement. One way to differentiate is that DILI usually presents as an asymptomatic abnormality in LFTs, which improves with the discontinuation of the drug. Imaging studies play a key role in excluding biliary obstruction, vascular abnormalities, and tumor-related causes, while liver biopsy is rarely required. However, in this case, a liver biopsy was performed, and histopathology revealed mild lobular inflammation, the most common finding in DILI, as well as bile duct loss in small portal tracts with associated portal inflammation and fibrosis. These features raised suspicion for VBDS, potentially representing a partial or early form of the disease. One of the elements of the diagnosis of VBDS is <50% of portal areas with bile duct in a biopsy with at least 10 portal areas in a sample taken at least a month after the onset of liver injury [10]. The limitation in this case is that the biopsy was performed within one week of the liver injury, as part of the initial workup to determine the cause of abnormal LFTs, rather than at the recommended interval. Despite this limitation, factors suggestive of VBDS are the following. First is the persistent elevation of ALP. Six months after the onset of DILI, her ALP remained elevated at 245 U/L (normal range: 53-141 U/L). Second, the patient tested negative for anti-mitochondrial antibody, which is highly sensitive and specific for primary biliary cholangitis. Third, she had no intrahepatic or extrahepatic biliary dilation noted on magnetic resonance cholangiopancreatography.

Identifying and discontinuing the offending drug was challenging due to her use of other hepatotoxic medications. Nevertheless, it was a crucial step in order to guide the next steps in her MM treatment plan. It is important to monitor for adverse events, including hepatotoxicity, during MM treatment, as well as to closely track disease progression while the patient is off therapy.

## Conclusions

This case is a rare presentation of lenalidomide-induced liver injury with histologic features of bile duct loss in small portal tracts, which may represent a partial or early form of VBDS. The limitations were the timing of the liver biopsy and the lack of a hepatopathologist in our hospital. Clinicians should be aware of this potential hepatotoxicity pattern, as early recognition may guide timely drug discontinuation and appropriate management.
